# Supplementary material for: Aerocavin is an antibiotic with potent and specific anti-Neisserial activity
Source: bioRxiv. 2026 Feb 23:2026.02.23.707490. Preprint. [Version 1] doi: 10.64898/2026.02.23.707490 (PMC12970324; doi:10.64898/2026.02.23.707490)
Supplement: Supplementary file 1 [file media-1.pdf]

Supplementary Tables

Table S1. *S. aureus* mutants resistant to aerocavin

**Isolate**	**Gene**	**Mutation**
GP1	*rpoC*	V1169E
GP2	*rpoB*	L1096F
GP3	*rpoB*	L1135F
GP4	*rpoC*	L1150F
GP5	*rpoC*	I1170T
GP6	*rpoB*	P1125L
GP7	*rpoC*	K334N
GP8	*rpoC*	K334N
GP9	*rpoC*	L1150F

Table S2. Mutations identified in aerocavin broth passages of *N. gonorrhoeae*

Line_Passage #	Gene	Mutation	Increase in MIC over the wildtype
1_28	*mtrR*	Y136*	8
	rpoB	**D1347Y**	
	rpoB	**T1305I**	
	spoT	D11A	
1_40	*rpoD*	P555S	32
	mtrR	G/A (promoter)	
	mtrR	Y136*	
	rpoB	**D1347Y**	
	rpoB	**T1305I**	
	spoT	D11A	
2_40	*pilQ*	V115fs	4
	spoT	N252K	
	mtrR	M1fs	
	atpB	C89fs	
3_40	*pilQ*	V162FS	8
	spoT	N252K	
	rpoB	**P1370L**	
	atpA	P280L	

Table S3. Strains and growth conditions used in the study

Species/strain	Propagation	MIC testing	Conditions
Chromobacterium sp. ATCC 53434	LB + Amp	CAMHB	30 °C
Neisseria gonorrhoeae (all strains)	CHOC	GC	37 °C, 5% CO_2
Staphylococcus aureus (all strains)	TS	CAMHB	37 °C
Escherichia coli K-12	LB	CAMHB	37 °C
Escherichia coli K-12 *tolC::Kan*	LB + Kan	CAMHB	37 °C
Acinetobacter baumannii ATCC 17978	LB	CAMHB	37 °C
Pseudomonas aeruginosa PA14	LB	CAMHB	37 °C
Klebsiella pneumoniae LM21	LB	CAMHB	37 °C
Proteus mirabilis ATCC 29006	LB	CAMHB	37 °C
Serratia marcescens M6C.2	LB	CAMHB	37 °C
Salmonella enterica ATCC 14028	LB	CAMHB	37 °C
Mycobacterium smegmatis mc^2 155	LB	CAMHB	37 °C
Enterococcus faecalis OG1RF	BHI	CAMHB	37 °C
Staphylococcus epidermidis ATCC 12228	BHI	CAMHB	37 °C
Chromobacterium violaceum ATCC 12472	LB + Amp	CAMHB	30 °C
Shewanella oneidensis MR-1	LB	CAMHB	30 °C
Vibrio cholerae V52	LB	CAMHB	37 °C
Burkholderia cenocepacia K56-2	LB	CAMHB	37 °C
Clostridium perfringens HN13	BHIS	BRU	37 °C, anaerobic
Clostridium difficile str. 630	BHIS	BRU	
Neisseria meningitidis ATCC 13077	CHOC	MHAB	37 °C, 5% CO_2
Bacillus subtilis 168	LB	CAMHB	37 °C

Table S4. Oligonucleotides used in the study

**Primer**	**Sequence**
acvA1_fwd	ccgaagctagcgaattcgtg-AAGGGCATCTCCGAGTGG
acvA1_rev	gttcccattt-TCGACTACCTTCCTCAATACAAAG
acvA2_fwd	aggtagtcga-AAATGGGAACGCTGCTCTTCC
acvA2_rev	aagccggctggcgccaagct-TGCCGCGTCATACTCGATG
pNPTS138_rev	CACGAATTCGCTAGCTTCGGC
pNPTS138_fwd	AGCTTGGCGCCAGCCGGC
pNPTS_sacB_PCR1	ACAACCATACGCTGAGAGATCC
pNPTS_sacB_PCR2	TTTTACAACGTCGTGACTGG
M13_pUC_fwd	CCCAGTCACGACGTTGTAAAACG
M13_pUC_rev	AGCGGATAACAATTTCACACAGG
acvA-seq1-1	CACCACCAACAACCGCATGG
acvA-seq4	ACAGCGTCATCGAGCCTATC

Supplementary Methods

Expression and purification of aerocavin

Chromobacterium sp. ATCC 53434 was grown at 30 °C with shaking at 200 rpm in 5 mL LB broth overnight. The cultures were then spread on the following medium (adapted from Singh *et al*[1]): 10 g/L yeast extract, 10 g/L malt extract, 1 g/L peptone, 20 g/L glucose, 15 g/L agar. The cultures were incubated at room temperature for five days, and then the agar was extracted three times with ethyl acetate. Ethyl acetate was removed by evaporation under vacuum, and the crude extract was solubilized in chloroform and extracted three times with water. Chloroform was removed by evaporation, and the extract was fractionated with C18 Sep-Pak columns (Waters) with stepwise acetonitrile gradient. The active fractions were further separated with Silica Sep-Pak columns (Waters) using stepwise hexanes/ethyl acetate gradient. The fraction containing the activity was injected into Phenomenex Synergi 4 µm Hydro-RP 80 Å, 250 x 10 mm preparative column holding 65% acetonitrile + 0.1% formate at 10 mL/minute flow rate. Purified aerocavin was stored at -20 °C in methanol.

NMR spectroscopy

The product's structure was confirmed with NMR. NMR spectral data were obtained by using a Bruker Avance 600 MHz spectrometer (Bruker), and the chemical shift values were represented in δ scales. Residual protium and carbon resonances of $CDCl_3$ NMR solvent were used to reference chemical shifts (δ_H 7.25 and δ_C 77.0). All the one-bond ^1H–^{13}C correlations were identified based on ^1H, ^{13}C, and HSQC NMR data, and the planar structure was assigned by detailed analysis of

COSY NMR spectrum along with interpretation of two- and three-bond $^{1}H-^{13}C$ HMBC correlations.

Paper disk activity screen

N. gonorrhoeae was grown overnight on chocolate agar, scraped, resuspended in Mueller Hinton broth, and spread with cotton-tipped applicators onto GCB agar supplemented with + 1% IsoVitaleX™ solution (BD). All other species were grown overnight in Mueller-Hinton broth and spread onto Mueller-Hinton Agar. Paper disks (Thermo) were impregnated with 100 µg of aerocavin and placed on top of the agar. *N. gonorrhoeae* was incubated at 37 °C in 5% CO_2, the other species were incubated under atmospheric conditions at 37 °C. *M smegmatis* was incubated for 48 hours, the other the species were incubated for 24 hours. To control for differences that media composition and atmospheric CO_2 concentration may have on aerocavin's activity, *S. aureus* Newman was additionally tested under the conditions used for *N. gonorrhea* to ensure that the results were comparable.

Determination of minimal inhibitory concentrations

Minimal inhibitory concentrations (MICs) were measured following CLSI guidelines[2-4]. MICs of *Neisseria* and *Clostridia* were determined using agar dilution assays[3, 4]. MICs of other bacteria were determined using broth macrodilution assays with agitation at 200 r. p. m. Table S3 contains information on media and conditions used for each strain. In all cases *S. aureus* Newman was included to control for large effects of differing conditions on aerocavin's activity. Sixty-four µg/mL was the highest concentration tested due to limited solubility of aerocavin.

Presence of aerocavin resistance-associated mutations in clinical *N. gonorrhoeae* isolates.

Publicly-available whole genome sequencing reads from 12,086 N. gonorrhoeae clinical isolates were downloaded from the European Nucleotide Archive[5-27]. Reads were assembled with SPAdes v 3.12.0[28] using the careful flag to correct assembled contigs. We additionally removed any contigs less than 500 nucleotides in length or with less than 10x coverage. We used blastn from BLAST+ v 2.9.0[29] to identify sequences of genes encoding the RNA polymerase in assembled genomes. Translated amino acid sequences were aligned with MAFFT v 7.402[30]. We characterized the diversity at each amino acid position previously identified to be associated with aerocavin resistance in other organisms.

Isolation of resistant mutants

S. aureus. Individual colonies were inoculated into tryptic soy broth (TSB) and incubated overnight at 37 °C with shaking at 200 rotations per minute (rpm). These cultures were then diluted 1/20 into liquid TSB containing 4X MIC of aerocavin and incubated overnight producing growth. The cultures were struck out on antibiotic-free media for acquisition of individual colonies and confirmed to retain aerocavin resistance after passages in antibiotic free-media.

N. gonorrhoeae. Colonies of resistant mutants were picked from the plates that were used to determine the frequency of resistance. The resistant mutants were re-streaked on agar containing aerocavin at the same concentration as that at which they were selected. To acquire isolates with secondary mutations, the procedure was repeated to isolate step 2 and step 3 mutants.

We did not isolate mutants from liquid passages of *N. gonorrhoeae*. Instead, passages 28 and 40 from line 1, and passages 40 from line 2 and 3 were spread from the frozen stock onto chocolate

agar plates and incubated overnight. The resulting biomass was collected for DNA isolation and sequencing.

DNA sequencing

DNA from resistant mutants was purified using Wizard® Genomic DNA Purification Kit (Promerga) or ZymoBIOMICS DNA Microprep Kit (Zymo Research) followed by cleanup with Genomic DNA Clean & Copncentrator-10 (Zymo Research). Illumina libraries were prepared using NEBNext® Ultra^{TM} DNA Library Kit (New England Biolabs) and sequenced on a MiSeq System with MiSeq Reagent v3 (2 x 300 bp) Kit (Illumina, Inc.). The data were analyzed using CLC Genomics software (QIAGEN).

RNA polymerase inhibition assay

RNA polymerase inhibition assay was carried out using *E. coli* RNA Polymerase Kit (Profoldin) and *E. coli* RNA Polymerase Holoenzyme (NEB). The assays were carried out in 384-well black flat bottom plates (Corning) adhering to the RNA Polymerase Kit instructions except RNAP purchased from NEB was used instead of the RNAP supplied with the kit. Prior to addition of the DNA template, the reactions were incubated with aerocavin or vehicle (methanol) for 5 minutes at 37 °C. The fluorescent readout of RNAP activity was measured on CLARIOstar multimode plate reader (BMG Biotech) with 485 nm excitation and 535 nm emission wavelengths. The data were analyzed using GraphPad Prism 10 using non-linear fit.

References

(1) Singh, P. D.; Liu, W. C.; Gougoutas, J. Z.; Malley, M. F.; Porubcan, M. A.; Trejo, W. H.; Wells, J. S.; Sykes, R. B. Aerocavin, a new antibiotic produced by Chromobacterium violaceum. *J Antibiot (Tokyo)* **1988**, *41* (4), 446-453. DOI: 10.7164/antibiotics.41.446 From NLM Medline.

(2) CLSI. *Performance Standards for Antimicrobial Susceptibility Testing. 31st ed. CLSI Supplement M100. Clinical and Laboratory Standards Institute*; 2021.

(3) CLSI. *Methods for Antimicrobial Susceptibility Testing of Anaerobic Bacteria. 9th ed. CLSI standard M11.* ; Wayne, PA, 2018.

(4) CLSI. *Methods for Dilution Antimicrobial Susceptibility Tests for Bacteria That Grow Aerobically. 11th ed. CLSI Standard M07*; Wayne, PA, 2018.

(5) Alfsnes, K.; Eldholm, V.; Olsen, A. O.; Brynildsrud, O. B.; Bohlin, J.; Steinbakk, M.; Caugant, D. A. Genomic epidemiology and population structure of Neisseria gonorrhoeae in Norway, 2016-2017. *Microb Genom* **2020**, *6* (4). DOI: 10.1099/mgen.0.000359 From NLM Medline.

(6) Buckley, C.; Forde, B. M.; Trembizki, E.; Lahra, M. M.; Beatson, S. A.; Whiley, D. M. Use of whole genome sequencing to investigate an increase in Neisseria gonorrhoeae infection among women in urban areas of Australia. *Sci Rep* **2018**, *8* (1), 1503. DOI: 10.1038/s41598-018-20015-x From NLM Medline.

(7) Cehovin, A.; Harrison, O. B.; Lewis, S. B.; Ward, P. N.; Ngetsa, C.; Graham, S. M.; Sanders, E. J.; Maiden, M. C. J.; Tang, C. M. Identification of Novel Neisseria gonorrhoeae Lineages Harboring Resistance Plasmids in Coastal Kenya. *J Infect Dis* **2018**, *218* (5), 801-808. DOI: 10.1093/infdis/jiy240 From NLM Medline.

(8) De Silva, D.; Peters, J.; Cole, K.; Cole, M. J.; Cresswell, F.; Dean, G.; Dave, J.; Thomas, D. R.; Foster, K.; Waldram, A.; et al. Whole-genome sequencing to determine transmission of Neisseria gonorrhoeae: an observational study. *Lancet Infect Dis* **2016**, *16* (11), 1295-1303. DOI: 10.1016/S1473-3099(16)30157-8 From NLM Medline.
(9) Demczuk, W.; Lynch, T.; Martin, I.; Van Domselaar, G.; Graham, M.; Bharat, A.; Allen, V.; Hoang, L.; Lefebvre, B.; Tyrrell, G.; et al. Whole-genome phylogenomic heterogeneity of Neisseria gonorrhoeae isolates with decreased cephalosporin susceptibility collected in Canada between 1989 and 2013. *J Clin Microbiol* **2015**, *53* (1), 191-200. DOI: 10.1128/JCM.02589-14 From NLM Medline.
(10) Demczuk, W.; Martin, I.; Peterson, S.; Bharat, A.; Van Domselaar, G.; Graham, M.; Lefebvre, B.; Allen, V.; Hoang, L.; Tyrrell, G.; et al. Genomic Epidemiology and Molecular Resistance Mechanisms of Azithromycin-Resistant Neisseria gonorrhoeae in Canada from 1997 to 2014. *J Clin Microbiol* **2016**, *54* (5), 1304-1313. DOI: 10.1128/JCM.03195-15 From NLM Medline.
(11) Eyre, D. W.; De Silva, D.; Cole, K.; Peters, J.; Cole, M. J.; Grad, Y. H.; Demczuk, W.; Martin, I.; Mulvey, M. R.; Crook, D. W.; et al. WGS to predict antibiotic MICs for Neisseria gonorrhoeae. *J Antimicrob Chemother* **2017**, *72* (7), 1937-1947. DOI: 10.1093/jac/dkx067 From NLM Medline.
(12) Ezewudo, M. N.; Joseph, S. J.; Castillo-Ramirez, S.; Dean, D.; Del Rio, C.; Didelot, X.; Dillon, J. A.; Selden, R. F.; Shafer, W. M.; Turingan, R. S.; et al. Population structure of Neisseria gonorrhoeae based on whole genome data and its relationship with antibiotic resistance. *PeerJ* **2015**, *3*, e806. DOI: 10.7717/peerj.806 From NLM PubMed-not-MEDLINE.

(13) Fifer, H.; Cole, M.; Hughes, G.; Padfield, S.; Smolarchuk, C.; Woodford, N.; Wensley, A.; Mustafa, N.; Schaefer, U.; Myers, R.; et al. Sustained transmission of high-level azithromycin-resistant Neisseria gonorrhoeae in England: an observational study. *Lancet Infect Dis* **2018**, *18* (5), 573-581. DOI: 10.1016/S1473-3099(18)30122-1 From NLM Medline.
(14) Grad, Y. H.; Harris, S. R.; Kirkcaldy, R. D.; Green, A. G.; Marks, D. S.; Bentley, S. D.; Trees, D.; Lipsitch, M. Genomic Epidemiology of Gonococcal Resistance to Extended-Spectrum Cephalosporins, Macrolides, and Fluoroquinolones in the United States, 2000-2013. *J Infect Dis* **2016**, *214* (10), 1579-1587. DOI: 10.1093/infdis/jiw420 From NLM Medline.
(15) Grad, Y. H.; Kirkcaldy, R. D.; Trees, D.; Dordel, J.; Harris, S. R.; Goldstein, E.; Weinstock, H.; Parkhill, J.; Hanage, W. P.; Bentley, S.; et al. Genomic epidemiology of Neisseria gonorrhoeae with reduced susceptibility to cefixime in the USA: a retrospective observational study. *Lancet Infect Dis* **2014**, *14* (3), 220-226. DOI: 10.1016/S1473-3099(13)70693-5 From NLM Medline.
(16) Harris, S. R.; Cole, M. J.; Spiteri, G.; Sanchez-Buso, L.; Golparian, D.; Jacobsson, S.; Goater, R.; Abudahab, K.; Yeats, C. A.; Bercot, B.; et al. Public health surveillance of multidrug-resistant clones of Neisseria gonorrhoeae in Europe: a genomic survey. *Lancet Infect Dis* **2018**, *18* (7), 758-768. DOI: 10.1016/S1473-3099(18)30225-1 From NLM Medline.
(17) Kwong, J. C.; Chow, E. P. F.; Stevens, K.; Stinear, T. P.; Seemann, T.; Fairley, C. K.; Chen, M. Y.; Howden, B. P. Whole-genome sequencing reveals transmission of gonococcal antibiotic resistance among men who have sex with men: an observational study. *Sex Transm Infect* **2018**, *94* (2), 151-157. DOI: 10.1136/sextrans-2017-053287 From NLM Medline.
(18) Lan, P. T.; Golparian, D.; Ringlander, J.; Van Hung, L.; Van Thuong, N.; Unemo, M. Genomic analysis and antimicrobial resistance of Neisseria gonorrhoeae isolates from Vietnam in

2011 and 2015-16. *J Antimicrob Chemother* **2020**, *75* (6), 1432-1438. DOI: 10.1093/jac/dkaa040 From NLM Medline.

(19) Lee, R. S.; Seemann, T.; Heffernan, H.; Kwong, J. C.; Goncalves da Silva, A.; Carter, G. P.; Woodhouse, R.; Dyet, K. H.; Bulach, D. M.; Stinear, T. P.; et al. Genomic epidemiology and antimicrobial resistance of Neisseria gonorrhoeae in New Zealand. *J Antimicrob Chemother* **2018**, *73* (2), 353-364. DOI: 10.1093/jac/dkx405 From NLM Medline.

(20) Mortimer, T. D.; Pathela, P.; Crawley, A.; Rakeman, J. L.; Lin, Y.; Harris, S. R.; Blank, S.; Schillinger, J. A.; Grad, Y. H. The Distribution and Spread of Susceptible and Resistant Neisseria gonorrhoeae Across Demographic Groups in a Major Metropolitan Center. *Clin Infect Dis* **2021**, *73* (9), e3146-e3155. DOI: 10.1093/cid/ciaa1229 From NLM Medline.

(21) Peng, J. P.; Yin, Y. P.; Chen, S. C.; Yang, J.; Dai, X. Q.; Zheng, H. P.; Gu, W. M.; Zhu, B. Y.; Yong, G.; Zhong, N.; et al. A Whole-genome Sequencing Analysis of Neisseria gonorrhoeae Isolates in China: An Observational Study. *EClinicalMedicine* **2019**, *7*, 47-54. DOI: 10.1016/j.eclinm.2019.01.010 From NLM PubMed-not-MEDLINE.

(22) Ryan, L.; Golparian, D.; Fennelly, N.; Rose, L.; Walsh, P.; Lawlor, B.; Mac Aogain, M.; Unemo, M.; Crowley, B. Antimicrobial resistance and molecular epidemiology using whole-genome sequencing of Neisseria gonorrhoeae in Ireland, 2014-2016: focus on extended-spectrum cephalosporins and azithromycin. *Eur J Clin Microbiol Infect Dis* **2018**, *37* (9), 1661-1672. DOI: 10.1007/s10096-018-3296-5 From NLM Medline.

(23) Sanchez-Buso, L.; Golparian, D.; Corander, J.; Grad, Y. H.; Ohnishi, M.; Flemming, R.; Parkhill, J.; Bentley, S. D.; Unemo, M.; Harris, S. R. The impact of antimicrobials on gonococcal evolution. *Nat Microbiol* **2019**, *4* (11), 1941-1950. DOI: 10.1038/s41564-019-0501-y From NLM Medline.

(24) Unemo, M.; Golparian, D.; Sanchez-Buso, L.; Grad, Y.; Jacobsson, S.; Ohnishi, M.; Lahra, M. M.; Limnios, A.; Sikora, A. E.; Wi, T.; et al. The novel 2016 WHO Neisseria gonorrhoeae reference strains for global quality assurance of laboratory investigations: phenotypic, genetic and reference genome characterization. *J Antimicrob Chemother* **2016**, *71* (11), 3096-3108. DOI: 10.1093/jac/dkw288 From NLM Medline.
(25) Thomas, J. C.; Seby, S.; Abrams, A. J.; Cartee, J.; Lucking, S.; Vidyaprakash, E.; Schmerer, M.; Pham, C. D.; Hong, J.; Torrone, E.; et al. Evidence of Recent Genomic Evolution in Gonococcal Strains With Decreased Susceptibility to Cephalosporins or Azithromycin in the United States, 2014-2016. *J Infect Dis* **2019**, *220* (2), 294-305. DOI: 10.1093/infdis/jiz079 From NLM Medline.
(26) Williamson, D. A.; Chow, E. P. F.; Gorrie, C. L.; Seemann, T.; Ingle, D. J.; Higgins, N.; Easton, M.; Taiaroa, G.; Grad, Y. H.; Kwong, J. C.; et al. Bridging of Neisseria gonorrhoeae lineages across sexual networks in the HIV pre-exposure prophylaxis era. *Nat Commun* **2019**, *10* (1), 3988. DOI: 10.1038/s41467-019-12053-4 From NLM Medline.
(27) Yahara, K.; Nakayama, S. I.; Shimuta, K.; Lee, K. I.; Morita, M.; Kawahata, T.; Kuroki, T.; Watanabe, Y.; Ohya, H.; Yasuda, M.; et al. Genomic surveillance of Neisseria gonorrhoeae to investigate the distribution and evolution of antimicrobial-resistance determinants and lineages. *Microb Genom* **2018**, *4* (8). DOI: 10.1099/mgen.0.000205 From NLM Medline.
(28) Bankevich, A.; Nurk, S.; Antipov, D.; Gurevich, A. A.; Dvorkin, M.; Kulikov, A. S.; Lesin, V. M.; Nikolenko, S. I.; Pham, S.; Prjibelski, A. D.; et al. SPAdes: a new genome assembly algorithm and its applications to single-cell sequencing. *J Comput Biol* **2012**, *19* (5), 455-477. DOI: 10.1089/cmb.2012.0021 From NLM Medline.

(29) Camacho, C.; Coulouris, G.; Avagyan, V.; Ma, N.; Papadopoulos, J.; Bealer, K.; Madden, T. L. BLAST+: architecture and applications. *BMC Bioinformatics* **2009**, *10*, 421. DOI: 10.1186/1471-2105-10-421 From NLM Medline.

(30) Katoh, K.; Standley, D. M. MAFFT multiple sequence alignment software version 7: improvements in performance and usability. *Mol Biol Evol* **2013**, *30* (4), 772-780. DOI: 10.1093/molbev/mst010 From NLM Medline.
